# Plants are visited by more pollinator species than pollination syndromes predicted in an oceanic island community

**DOI:** 10.1038/s41598-020-70954-7

**Published:** 2020-08-18

**Authors:** Xiangping Wang, Meihong Wen, Xin Qian, Nancai Pei, Dianxiang Zhang

**Affiliations:** 1 Key Laboratory of Plant Resources Conservation and Sustainable Utilization, South China Botanical Garden, Chinese Academy of Sciences, Guangzhou, 510650 Guangdong China; 2 Research Institute of Tropical Forestry, Chinese Academy of Forestry, Guangzhou, China

**Keywords:** Plant ecology, Plant evolution, Plant reproduction

## Abstract

The pollination syndrome concept has provided powerful utility in understanding the evolution and adaptation of floral traits. However, the utility of this conception has been questioned on the grounds that flowers usually attract a broader spectrum of visitors than one might expect. Furthermore, the relationship between plant specialization and floral traits is poorly understood. Here, we examined the applicability of using the pollination syndrome to predict the pollinators of plants on Yongxing Island. We used the species-level specialization of pollination networks to compare the difference of plant ecological specialization among floral traits. The result of full model was not significant, indicating that floral traits did not affect the pollinator functional groups. The five floral traits explained only 22.5% of the pollinator’s visitation preference. Our results showed that plants were visited by more pollinator species than pollination syndromes predicted. Plants with restrictive flowers showed higher specialization than those with unrestrictive flowers, while other floral traits exhibited no significant effect on plant specialization. Generalized pollination system on oceanic island might influence the predictive accuracy of pollination syndromes and the relationship between floral traits and plant ecological specialization. Our findings highlighted the utility and limitations of pollination syndromes concept in oceanic island communities.

## Introduction

The pollination syndrome concept implies that plants specialize on particular functional groups of pollinators that exert similar selective pressures on floral traits^1^. Thus, flowers pollinated by the same functional group of pollinators are expected to converge onto similar phenotypes in response to selection imposed by the pollinators^2,3^. Many studies have found support for the pollination syndromes concept^4–10^, suggesting that convergent evolution of floral traits is driven mainly by adaption to the effective pollinator functional group across angiosperms^11^. However, other studies still caution its utility and predictability^12–20^. Furthermore, plant-pollinator interactions have proved to be more generalized than was previously thought in nature, thus, the utility of pollination syndromes in predicting the specialization in pollination system and the association of particular floral traits with specific pollinators have been questioned^1,21–24^. Although the usefulness of pollination syndromes has been extensively studied in different plant groups and geographic regions across the world, the debate about the reliability of pollination syndromes is ever existing.

Additionally, differences in geographical distribution may influence the relationship between plants and pollinators. The latitudinal gradient in species diversity has been associated with a greater strength of biotic interactions at lower than at higher latitudes^25^. Empirical studies using mutualistic plant-pollinator networks have found higher specialization in the tropics^23,26^, although Ollerton and Cranmer^27^ still regards the viewpoint debatable. Under this scenario, stronger biotic interactions might lead to greater species diversification and coexistence in tropical regions^25,28^. Therefore, the predictability of pollination systems and pollination mode from floral syndromes should be greater in the tropics than in other regions^11^. In the other aspect, however, compared with the mainland, islands normally have lower species richness with fewer pollinator species^5,29–31^, and plants on islands often depend on more than one type of pollinator^32^. Thus, a community level test of pollination syndromes on oceanic islands is needed. Such an attempt would provide us new evidence about the use of classical pollination syndromes to classify plant-pollinator interactions^33^.

Plant-pollinator interactions in oceanic island systems are predominantly generalized compared with continental communities, likely as a result of more depauperate and disharmonic pollinator faunas^22,34–39^, which provides a direct test of the impact of a change in the relative abundance of pollinator functional groups on the relationship between floral traits and pollinators. Furthermore, island plants tend to have smaller, less brightly colored floral displays, morphologically unspecialized with more bowl-shaped corollas compared with their mainland relatives^40,41^. Self- compatibility species are over-represented on islands, which may also influence the floral traits evolution^42^. Several studies have shown that floral traits can lead to accurate predictions about the effective pollinators of plant species^7,11,43,44^. Thus, on islands, these characteristics of flowers may have been influenced by the presence of pollinator groups different from their continental counterparts in terms of morphology and behavior^45^. In such case, plants should generalize on a wide range of pollinators, and such ecological generalization is indeed frequently found^36,37^. Nevertheless, whether the predictability of pollination syndromes on oceanic islands is still warrant remains unknown. A few studies have focused on the bird pollination syndrome on islands, for example, Biddick and Burns^10^ found that the phenotypic trait matching between birds and flowering plants supported the pollination syndromes. Whereas plants evolved generalist bird pollination as an adaptation to birds as a reliable pollinator group^46^, and bird-visited flowers display mixed traits not fitting the classical ornithophilous syndrome on islands^24^. Therefore, studies dealing with the relationship between floral traits and different pollinator functional groups in oceanic island communities are urgently needed.

This study aimed to test the applicability and predictability of the pollination syndromes in a relatively generalized pollination system of an oceanic island. We assessed whether floral traits predicted the major pollinators by analyzing the composition of different pollinators to plant species, and by including measures of ecological specialization. Specifically, we used data on 55 plant species mostly native to, or in a few cases naturalized in, the Yongxing Island community to test the validity of the pollination syndrome concept by addressing how floral traits relate to pollinator species. We further explored the association between floral traits (evolutionary specialization) and ecological specialization levels of plant species by calculating the species-specific indices (d' index) of pollination network. To the best of our knowledge, this is the first community-wide study assessing the pollination syndromes on an oceanic island community covering different pollinator groups.

## Materials and methods

### Study site and community

**Figure 1:**
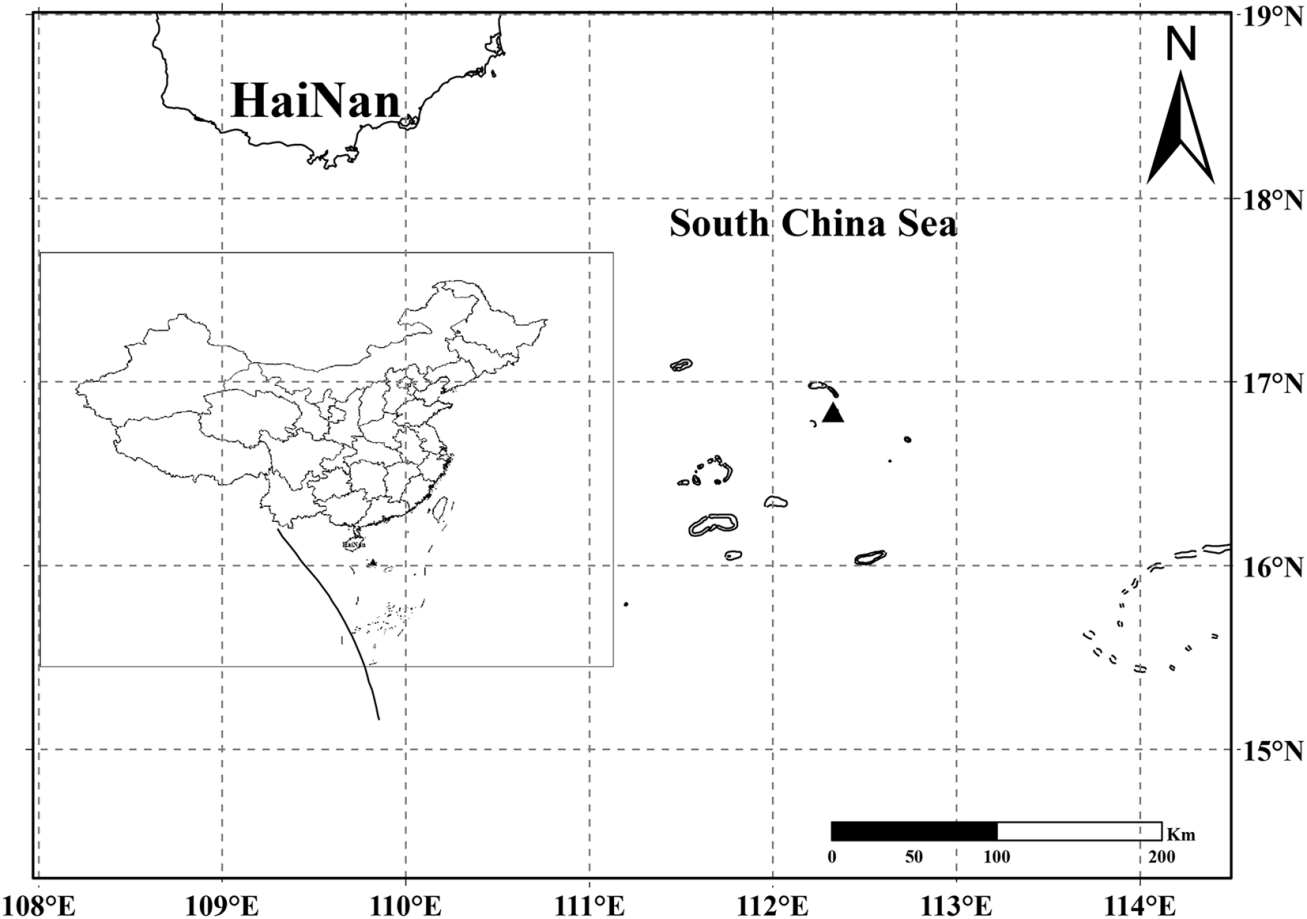
Location of Yongxing Island (black triangle).

The Paracel Islands (Xisha Islands) are a series of coral islets, locating in South China Sea. The Yongxing Island (16° 50.1′ N, 112° 19.8′ E), with a total area of 2.6 km^2^, is the largest islet of this archipelago (Fig. 1). The islet shows a typical tropical oceanic climate, with no apparent difference in flowering pattern around the year. We conducted our study within sample plots of typical plant communities in the island, which include tree, shrub, and herb species such as *Cordia subcordata*, *Scaevola taccada*, *Tribulus cistoides*, from early April to early June in 2017.

### Field surveys and identification of pollinator species

We conducted 20 quadrats with size of 5 × 5 m^2^ in a systematic way, at least 5 m away from each other on the island. We assessed visitation to flowers inside 20 quadrats using 30-min observation periods. To collect enough information on the visits to all the flowering plant species, and to minimize the statistic errors caused by the sampling strategy, we randomly changed the observation orders of these quadrats within a day after each observation period^4^. The observations were conducted on sunny days without wind when the weather conditions were suitable for pollinator activity from 8:00 to 19:00 h. The order of observation of quadrats was random, and we did not observe the same quadrat more than once per day. During each observation period we collected floral visitors from each insect-pollinated species by using sweep net. We observed at least 20 h in all for each quadrat. Only those flower visitors whose body actually touched the reproductive organs (stigmas and/or stamens) for more than one second foraging for nectar and/or pollen were classified as legitimate visitors and collected^15,47^, and this visit was defined to have occurred effectively.

All the visitors were firstly morphotyped and 3–10 individuals of each morphotype were collected for further identification to the lowest possible taxonomic level by entomologists (see acknowledgments). To verify if the flower visitors were the potential pollinators, we collected another five individuals of the same species which visited flowers of the same plant species and examined insect body pollen loads for pollen analysis. Pollen sample of each visitor species was checked under a JSM-6360LV scanning electron microscope. Pollen grains were identified by comparing with a reference library of pollen based on those removed from field-collected flowers. If more than 80% specimens of samples carried the host plant pollen grains, we presumed that the visitor was a potential pollinator (hereafter, named pollinator), otherwise, we presumed this visitor as ineffective pollinator and the insect was excluded for data analysis^48^. Because of the depauperate pollinator fauna on Yongxing Island, we categorized each pollinator into eight functional groups, i.e. clusters of pollinator species that behave in a similar way in the flowers and might therefore exert similar selective pressures^1^. The functional groups categorized are: Apidae, non-Apidae Hymenoptera, Syrphidae (hover flies), non-Syrphidae Diptera, Butterflies, Hawkmoths, Hemiptera (Triatominae) and Passeriformes (*Zosterops japonicus*). To avoid overestimating specialization levels, we only included in the analyses those plant species (55 plant species) that received at least five visits during the whole study period^12^. Voucher specimens of plants and pollinators were deposited in the South China Botanical Garden, Chinese Academy of Sciences, Guangzhou.

### Measurements of floral traits

We used six floral traits to describe the plant species^4^: (1) floral symmetry, by characterizing species as radial and bilateral symmetry based on corolla or inflorescences (e.g. Asteraceae) architecture^15^. Radial symmetry flowers or inflorescences are held erect, have easily accessible floral rewards in bowl-shaped or trumpet perianths. In bilateral symmetry flowers, nectar or/and pollen grain are hidden within the corollas and only selective foragers are able to access. (2) Floral restrictiveness, by distinguishing as having a restrictive or unrestrictive morphology according to the complexity of accessibility for visitors^49^. Flowers have easily accessible floral rewards with nectar or/and pollen exerted beyond the petals as unrestrictive. Flowers with a tube radius of at least 2 mm as unrestrictive and less than 2 mm as restrictive, as the smaller openings could prevent access by visitors lacking elongate proboscises. (3) Floral color, as seen by humans-white, yellow, pink, purple, orange, red and blue, using color classes similar to those used by previous related studies^49,50^, although color can be measured by providing a reflectance spectrum^51^. Flowers with more than one color are classified according to the predominant color. (4) Flower clustering, by distinguishing between species with solitary flowers and those with inflorescences^4^. (5) Corolla tube length, as measured in the field (mean value per species based on measurements of 20 flowers from 20 individuals; species with open, radial symmetry flowers received a tube length of zero. (6) Flower size (the calculation method is shown below).

To estimate the flower size of each plant species, twenty completely opened flowers randomly selected from 20 individuals were measured with a digital caliper in the field. Following Hegland and Totland’s^15^ method, flower size (display area size) of each species was calculated according to the flower or inflorescences shape. We classified flowers into five types: (1) For flowers with circular outline, shallow or flat flowers, and the capitulum as a circle (e.g.* Tribulus cistoides*), we calculated the size using the formula πr^2^, where r is the radius of the flower; (2) For bilateral symmetry flowers (e.g.* Cleome viscosa*), we used the formula L × W, where L and W are the length and width of the square-shaped flat corolla; (3) For flowers with both flat corolla and tubular structure, we used the formula L × W + πBD (e.g. *Stachytarpheta jamaicensis*) or πr^2^ + πBD (e.g.* Guettarda speciosa*), where L and W are the length and width of the square-shaped flat corolla, B is the bore diameter of the tubular structure according to its shape, D is the corolla tube length; (4) For spherical flowers or inflorescence (e.g.* Mimosa pudica*), we used the formula 4πr^2^, where r is the radius of the sphere; (5) For flowers from leguminous plants, we used the formula L × W + l × w, where L and W are the length and width of the banner, and l and w are the length and width of the wing (see Table 1 for formulate used for each plant species). For Asteraceae, Euphorbiaceae and Leguminosae (*Mimosa pudica*), we used display size of an inflorescence.

**Table 1:** Basic characteristics [their family, life form (Lf), floral symmetry (Sy), floral color, floral restrictiveness (Re), flower clustering (Infl)], corolla tube length (mm) (CTL) and flower size (mm^2^) of the 55 plant species in the Yongxing Island community.

Study species	Family	Lf	Sy	Color	Re	Infl	CTL	Formula	Flower size
*Abutilon indicum*	Malvaceae	Herb	R	Yellow	No	No	0	πr^2^	492.53 ± 15.93
*Bidens pilosa*	Asteraceae	Herb	R	White	No	Yes	2.4	πr^2^	488.51 ± 25.51
*Boerhavia diffusa*	Nyctaginaceae	Herb	R	Pink	Yes	Yes	0	πr^2^	2.36 ± 0.08
*Bougainvillea spectabilis*	Nyctaginaceae	Shrub	R	White	Yes	Yes	19.4	πr^2^ + πBD	132.67 ± 2.89
*Canavalia maritima*	Fabaceae	Herb	B	Purple	Yes	Yes	0	L × W + l × w	861.30 ± 18.54
*Carica papaya*	Caricaceae	Tree	R	White	Yes	Yes	20.6	πr^2^ + πBD	625.85 ± 15.77
*Catharanthus roseus*	Apocynaceae	Herb	R	Pink	Yes	Yes	30.3	πr^2^ + πBD	1978.66 ± 83.86
*Chromolaena odorata*	Asteraceae	Herb	R	Purple	No	Yes	2.2	πr^2^	200.62 ± 6.21
*Cleome viscosa*	Capparidaceae	Herb	B	Yellow	No	No	5.4	L × W	203.95 ± 6.37
*Clerodendrum inerme*	Verbenaceae	Shrub	B	White	Yes	Yes	32.3	L × W + πBD	648.56 ± 17.05
*Coccinia grandis*	Cucurbitaceae	Herb	R	White	No	No	11.6	πr^2^ + πBD	1,530.88 ± 70.39
*Colubrina asiatica*	Rhamnaceae	Shrub	R	Yellow	No	Yes	0	πr^2^	28.49 ± 0.62
*Cordia subcordata*	Boraginaceae	Tree	R	Orange	No	Yes	27.3	πr^2^ + πBD	2,235.12 ± 71.78
*Crotalaria pallida*	Fabaceae	Herb	B	Yellow	Yes	Yes	0	L × W + l × w	128.47 ± 1.77
*Datura metel*	Solanaceae	Herb	R	White	No	No	168.2	πr^2^ + πBD	33,342.71 ± 900.19
*Eclipta prostrata*	Asteraceae	Herb	R	White	No	Yes	1.2	πr^2^	50.40 ± 2.82
*Euphorbia atoto*	Euphorbiaceae	Herb	R	Yellow	No	Yes	0	πr^2^	187.95 ± 11.62
*Euphorbia cyathophora*	Euphorbiaceae	Herb	R	Red	No	Yes	0	πr^2^	2,333.94 ± 140.20
*Euphorbia hirta*	Euphorbiaceae	Herb	R	Yellow	No	Yes	0	πr^2^	34.41 ± 1.82
*Gossypium hirsutum*	Malvaceae	Shrub	R	Yellow	No	No	0	πr^2^ + πBD	4,412.09 ± 242.66
*Guettarda speciosa*	Rubiaceae	Tree	R	White	Yes	Yes	35.5	πr^2^ + πBD	1,069.42 ± 41.84
*Herissantia crispa*	Malvaceae	Herb	R	Yellow	No	No	0	πr^2^	108.79 ± 3.03
*Ipomoea obscura*	Convolvulaceae	Herb	R	White	No	Yes	14.4	πr^2^ + πBD	891.95 ± 50.36
*Ipomoea pescaprae*	Convolvulaceae	Herb	R	Purple	No	Yes	26.0	πr^2^ + πBD	4,454.55 ± 120.83
*Ixora chinensis*	Rubiaceae	Shrub	R	Red	Yes	Yes	34.5	πr^2^ + πBD	539.412 ± 13.45
*Lantana camara*	Verbenaceae	Shrub	R	Red	Yes	Yes	11.3	πr^2^ + πBD	101.28 ± 2.73
*Macroptilium atropurpureum*	Fabaceae	Herb	B	Purple	Yes	Yes	0	L × W + l × w	427.63 ± 10.09
*Messerschmidia argentea*	Boraginaceae	Shrub	R	White	No	Yes	0	πr^2^	22.88 ± 1.55
*Mimosa pudica*	Fabaceae	Herb	R	Pink	No	Yes	0	4πr^2^	1,186.99 ± 20.78
*Morinda citrifolia*	Rubiaceae	Tree	R	White	No	Yes	9.9	πr^2^ + πBD	260.74 ± 5.60
*Passiflora foetida*	Passifloraceae	Herb	R	White	No	No	0	πr^2^	1,073.74 ± 27.20
*Phyla nodiflora*	Verbenaceae	Herb	B	White	Yes	Yes	2.0	πr^2^	4.21 ± 0.15
*Physalis minima*	Solanaceae	Herb	R	Yellow	No	No	4.3	πr^2^ + πBD	142.11 ± 5.58
*Pisonia grandis*	Nyctaginaceae	Tree	R	White	No	Yes	0	πr^2^	20.35 ± 0.79
*Portulaca grandiflora*	Portulacaceae	Herb	R	Pink	No	No	0	πr^2^	859.76 ± 30.21
*Portulaca oleracea*	Portulacaceae	Herb	R	Yellow	No	No	0	πr^2^	37.89 ± 1.48
*Rhynchosia minima*	Fabaceae	Herb	B	Yellow	Yes	Yes	0	L × W + l × w	57.13 ± 1.00
*Ricinus communis*	Euphorbiaceae	Shrub	R	White	No	Yes	0	4πr^2^	198.89 ± 11.02
*Scaevola taccada*	Goodeniaceae	Shrub	B	White	No	Yes	17.5	L × W	405.70 ± 25.45
*Senna occidentalis*	Fabaceae	Shrub	B	Yellow	No	Yes	0	L × W + l × w	519.12 ± 19.97
*Sesbania cannabina*	Fabaceae	Shrub	B	Yellow	Yes	Yes	0	L × W + l × w	147.55 ± 3.06
*Sesuvium portulacastrum*	Aizoaceae	Herb	R	Pink	No	No	0	πr^2^	147.99 ± 5.64
*Sida alnifolia*	Malvaceae	Herb	R	Yellow	No	No	0	πr^2^	136.67 ± 6.52
*Solanum photeinocarpum*	Solanaceae	Herb	R	White	No	Yes	0	πr^2^	47.36 ± 2.91
*Stachytarpheta jamaicensis*	Verbenaceae	Herb	B	Blue	Yes	Yes	10.2	L × W + πBD	177.06 ± 2.33
*Suriana maritima*	Simaroubaceae	Tree	R	Yellow	No	Yes	0	πr^2^	102.70 ± 1.23
*Terminalia catappa*	Combretaceae	Tree	R	White	No	Yes	0	πr^2^	45.48 ± 3.28
*Trianthema portulacastrum*	Aizoaceae	Herb	B	Pink	No	No	0	L × W	54.75 ± 4.37
*Tribulus cistoides*	Zygophyllaceae	Herb	R	Yellow	No	No	0	πr^2^	577.82 ± 40.05
*Tridax procumbens*	Asteraceae	Herb	R	White	No	Yes	4.8	πr^2^	158.52 ± 5.00
*Triumfetta procumbens*	Tiliaceae	Herb	R	Yellow	No	Yes	0	πr^2^	385.38 ± 19.84
*Vernonia cinerea*	Asteraceae	Herb	R	Pink	No	Yes	0	πr^2^	15.29 ± 0.67
*Vigna marina*	Fabaceae	Herb	B	Yellow	Yes	Yes	0	L × W + l × w	396.69 ± 9.11
*Wedelia biflora*	Asteraceae	Herb	R	Yellow	No	Yes	3.0	πr^2^	590.48 ± 51.13
*Wedelia trilobata*	Asteraceae	Herb	R	Yellow	No	Yes	3.3	πr^2^	843.53 ± 31.06

‘Flower formulae’ for the measurement of area of floral visual units: πr^2^ (r = corolla radius) for flowers with circular outlines; L × W (L = corolla length, W = corolla width) for bilaterally symmetrical with flat flowers; L × W + πBD (L = flat corolla length, W = flat corolla width, B = bore diameter, D = depth of tubular part) or πr^2^ + πBD (r = radius of circular part, B = bore diameter, D = depth of tubular part) for flowers had both tubular structure and flat corolla; 4πr^2^ ( r = sphere radius) for spherical flowers and L × W + l × w (L = length, W = width of banner, l = length, w = width of wing) for leguminous plants.

### Plant specialization indices

To assess the role of each plant species within networks, we constructed a bipartite plant-pollinator network of the community by combining data of field observation and pollen analysis of potential pollinators. We calculated the species-level specialization index d' which measures the degree of a plant’s specialization on flower-visiting taxa, with higher values indicating higher specialization in a community^52–54^. The d' index is appropriate to compare specialization of species within networks because it has the advantage of not being affected by network size and sampling intensity^53^. The d' index is derived from Kullback–Leibler distance (as is Shannon’s diversity index), and calculates how strongly a species deviates from a random sampling of interacting partners available. It ranges from 0 (no specialization) to 1 (perfect specialist). In the case of a pollination web, a pollinator may be occurring only on one plant species, but if this species is the most dominant one, there is limited evidence for specialization. Hence this pollinator would receive a low value. In contrast, a pollinator that occurs only on the two rarest plants would have a very high value of d'. Calculations of the index d' were conducted with the “bipartite” package^55^ in R version 3.4.4^56^.

### Statistical analysis

We checked for multicollinearity between the fixed factors using a Variation Inflation Factor (VIF) test, assuming VIF > 3 as benchmark for collinearity^57^. Only corolla tube length and flower size were correlated (VIF > 9), so we chose to use only flower size in analyses. We investigated the relationships between floral traits and pollinators by conducting multivariate analyses. To decide the ordination method of our data, we first conducted a detrended correspondence analysis (DCA). The long gradient length was < 2, indicating that linear models best captured the variance in the data. Therefore, we used redundancy analysis (RDA, vegan package in R version 3.4.4.) to test the relationship between floral traits and pollinator functional group composition in our community. The sample units were the plant species in the community. We used the number of potential pollinator species of each functional group to a particular plant species as the response variable, and the floral traits as the explanatory variable. We used 999 Monte Carlo permutations to assess statistical significance of the association between the identity of potential pollinators and floral traits. First, we tested the significance of the full model. Second, we tested separately the effects of each floral traits on the identity of pollinator visits.

To examine the relationship between floral traits and the ecological specialization level (d'), we used the analysis of covariance (ANCOVA) (SPASS 19.0). The index of d' was the response variable and floral symmetry, floral color, floral restrictiveness and flower clustering were the predictor fixed variables. The flower size was entered as a covariable. Because the corolla tube length was correlated to flower size, thus we did not include corolla tube length in the analysis. We used Tukey’s a posteriori tests to conduct multiple comparisons among levels of significant factors in the ANCOVA, and took the overall variance structure into account.

## Results

In total, 55 species (in 23 families) of plants were observed to be insect-pollinated, 57 insect species were pollinators in the community. The relative proportions of each floral trait and each pollinator functional group were shown in Tables 2 and 3, respectively. Both plants and pollinators had abundant linkages as shown by that per plant species have an average of 7.1 pollinator species, and per pollinator species visited an average of 9.1 plant species. The most generalized pollinators were Apidae, which visited on average 21.5 plant species. Flowering plants and the numbers of their pollinator species to each functional group can be found as Supplementary Table S1 online.

**Table 2:** The relative proportion of each floral trait in 55 plant species in Yongxing Island community.

Flora trait	Proportion (%)
**Floral symmetry**	
Radial	76.4
Bilateral	23.6
**Floral restrictiveness**	
Restrictive	30.9
Unrestrictive	69.1
**Flower clustering**	
Inflorescences	74.5
Solitary flowers	25.5
**Floral color**	
Yellow	36.4
White	34.5
Pink	12.7
Purple	7.3
Red	5.5
Orange	1.8
Blue	1.8

**Table 3:** The relative proportion of each pollinator functional group in 57 animal species in Yongxing Island community.

Pollinator taxa	Functional group	Proportion (%)
Hymenoptera	Apidae	10.5
	Non-apidae Hymenoptera	35.1
Diptera	Syrphidae	7.0
	Non-syrphidae Diptera	17.6
Lepidoptera	Butterflies	19.3
	Hawkmoths	7.0
Hemiptera	Triatominae	1.75
Passeriformes	Zosteropidae	1.75

**Figure 2:**
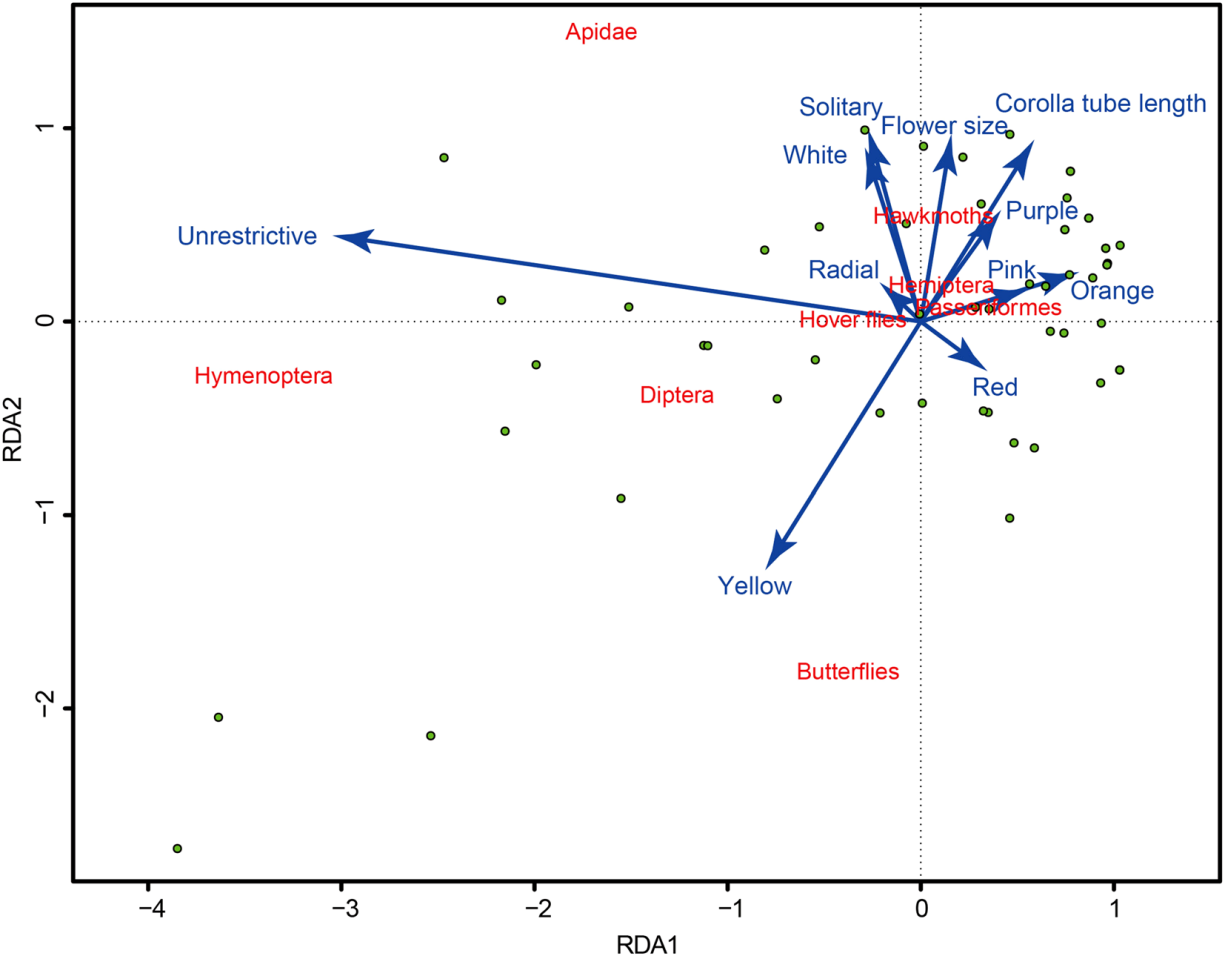
Redundancy analysis (RDA) conducted for the composition of pollinators and the floral traits in Yongxing Island community. Variables are indicated on each arrow; direction of arrows indicates the sense of correlations. Green filled circles represent plant species. Refer to Table 3 for explanations on variables.

The result of full model (RDA) was not significant (*df* = 10, Inertia = 7.733, F = 1.134, *P* = 0.343), indicating that these floral traits did not have a significant effect on the composition of pollinators. The five floral traits explained only 22.5% of the pollinator’s visitation preference for flowers. However, among all the separate RDA ordinations, flower restrictiveness had a significant effect (R^2^ = 0.082, *P* = 0.026) on the composition of pollinators to each plant in the community (Table 4). Floral symmetry, flower color, flower clustering and flower size did not have significant effects on the composition of pollinators to each plant species (Table 4). The distance among pollinator functional group and floral traits in the ordinations indicates how closely they are associated (Fig. 2). In the community, the proportion of Hymenoptera pollinators was higher for the plant species with unrestrictive flowers (Fig. 2). While other pollinator functional groups had no preference on the selectiveness of flowers (Fig. 2).

**Table 4:** Results of the redundancy analyses used to test the relationship between floral traits and pollinators.

Floral trait	Variable	RDA1	RDA2	R^2^	*P*
Flower clustering	Inflorescences	0.039	− 0.247	0.026	0.252
	Solitary	− 0.113	0.723		
Floral symmetry	Bilateral	0.093	− 0.155	0.001	0.922
	Radial	− 0.029	0.048		
Floral color	Blue	0.530	0.169	0.067	0.751
	Orange	1.462	0.799		
	Pink	0.360	0.190		
	Purple	0.360	0.875		
	Red	0.349	− 0.455		
	White	− 0.097	0.526		
	Yellow	− 0.258	− 0.721		
Floral restrictiveness	Restrictive	1.103	− 0.282	0.082	**0.026**
	Unrestrictive	− 0.493	0.126		
Flower size		− 0.466	0.885	0.060	0.134
Corolla tube length		− 0.245	0.969	0.045	0.203

*P* < 0.05 shown in bold.

**Figure 3:**
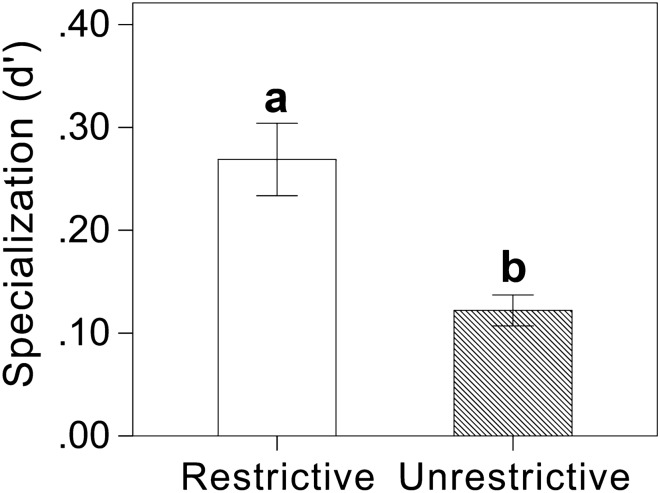
Comparisons of plants’ specialization (d') between plants with restrictive flowers and plants with unrestrictive flowers in Yongxing Island community. Different letters represented significant difference at *P* ≤ 0.01 level based on ANCOVA analysis.

The floral symmetry (*df* = 1, F = 0.731, *P* = 0.399), flower color (*df* = 6, F = 1.256, *P* = 0.303), and flower clustering (*df* = 1, F = 0.021, *P* = 0.886) had no effect on the specialization (d') of flowers. Flower size was not significantly related to the specialization level of flowers (*df* = 1, F = 0.035, *P* = 0.852). Floral restrictiveness (*df* = 1, F = 8.967, *P* = 0.005) strongly affected specialization levels of flowers, and plant species with restrictive flowers (d' = 0.27 ± 0.04) showed a higher degree of specialization than that of unrestrictive flowers (d' = 0.12 ± 0.01) (Fig. 3). All other interactions were not significant (all *P* >> 0.05). Significance of the whole model was R^2^ = 0.023, *df* = 20, F = 1.698, *P* = 0.085.

## Discussion

**Figure 4:**
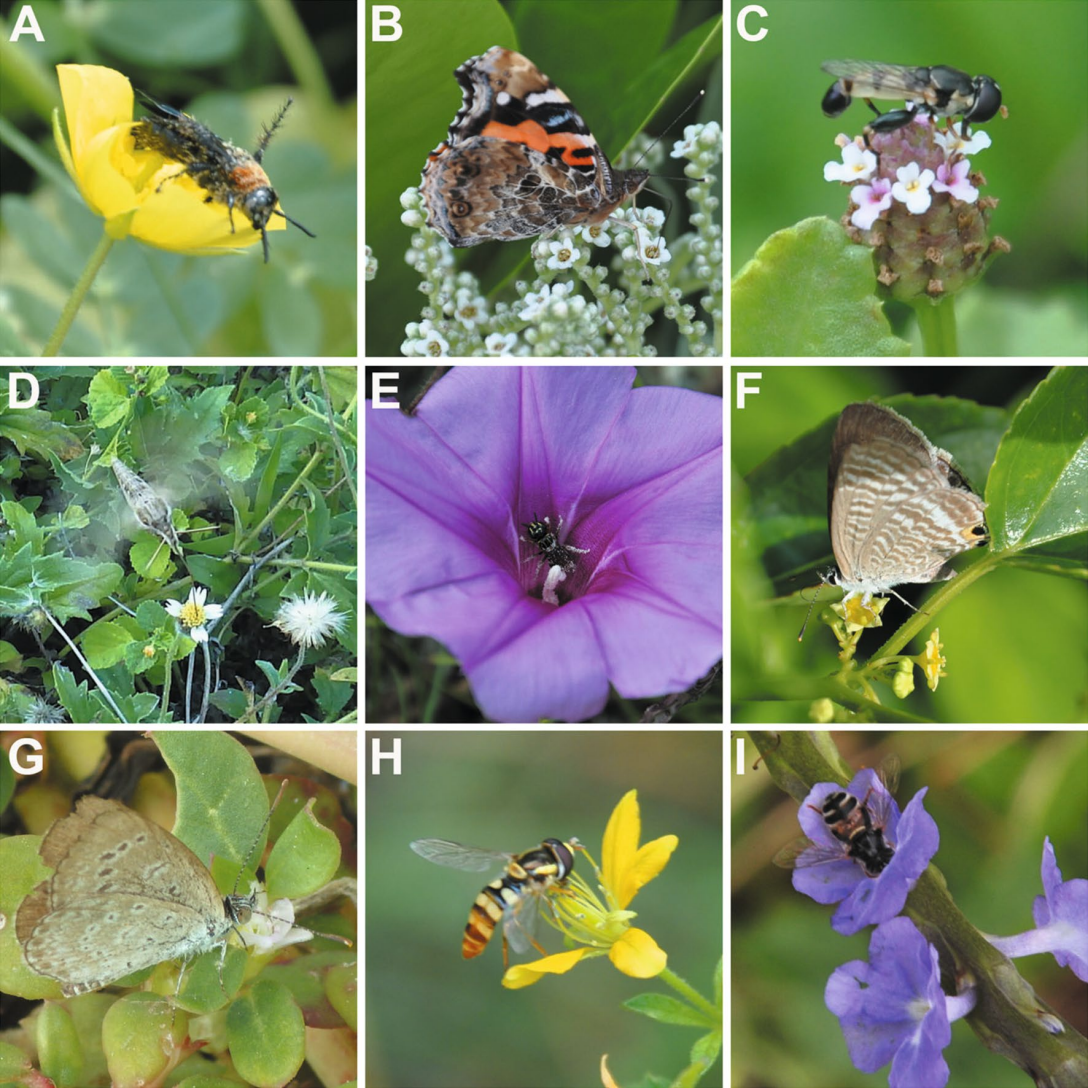
Examples of interactions that plants were visited by more pollinator species than pollination syndromes predicted in Yongxing Island community. (**A)** *Campsomeriella collaris* visiting *Tribulus cistoides*; (**B**) *Vanessa indica* visiting *Messerschmidia argentea*; (**C**) *Syritta orientalis* visiting *Phyla nodiflora*; (**D**) *Hippotion velox* visiting *Tridax procumbens*; (**E**) *Ceratina lieftincki* visiting *Ipomoea pescaprae*; (**F**) *Lampides boeticus* visiting *Colubrina asiatica*; (**G**) *Zizina Otis* visiting *Trianthema portulacastrum*; (**H**) *Ischiodon scutellaris* visiting *Cleome viscosa*; (**I**) *Paragus bicolor* visiting *Stachytarpheta jamaicensis*.

In our study, the results showed that floral traits did not significantly affect the pollinator functional groups, and we did not find significant correlation among floral traits and main pollinators, i.e., the pollination syndrome concept was not supported. Further, our results showed that plants were visited by more pollinator species than pollination syndromes predicted. Specifically, Apidae pollinated most of the plant species (85.5% of all the observed plant species) including the flowers predicted by the classic pollination syndrome concept^58^, and not visited flowers with long corolla tube and white colors (e.g., *Bougainvillea spectabilis*, *Clerodendrum inerme* and *Guettarda speciosa*). Non-apidae Hymenoptera pollinated 63.6% of all the observed plant species including bilateral symmetry flowers with pink or purple flowers which were predicted by the classic pollination syndrome concept^58^. They not only pollinated flowers with above-mentioned floral traits but also pollinated radial symmetry flowers with yellow or white colors (e.g., Asteraceae, *Tribulus cistoides*, *Colubrina asiatica*, *Messerschmidia argentea* and *Passiflora foetida*). Lázaro et al.^4^ found that hover flies were associated with radial symmetry flowers in two lowland communities whereas visited bilateral symmetry in alpine community. However, in our community, hover flies pollinating radial symmetry flowers (e.g., *Bidens Pilosa*, *Boerhavia diffusa* and *Chromolaena odorata*) and bilateral symmetry flowers (e.g., *Cleome viscosa*, *Phyla nodiflora* and *Stachytarpheta jamaicensis*) simultaneously. Butterflies not only pollinated flowers with large and showy, pink in color, and long corolla tube (e.g., *Bougainvillea spectabilis*, *Catharanthus roseus*, *Ixora chinensis* and *Lantana camara*), but also pollinated Asteraceae and Fabaceae with white or yellow flowers, and radial symmetry flowers with yellow color without corolla tube (e.g., *Tribulus cistoides* and *Trianthema portulacastrum*). Based on the pollination syndrome concept, hawkmoths visited flowers tend to be white, night-opening, large and showy with tubular corollas. In our study, hawkmoths pollinated flowers with the above-mentioned shape and other flowers without corolla tube (*Trianthema portulacastrum*) and Asteraceae (e.g. *Tridax procumbens*). Hemiptera (Triatominae) mainly pollinated Asteraceae, and the Passeriformes (*Zosterops japonicus*) only foraged on flowers of *Cordia subcordata* with tubular corolla and orange color which was consistent with the syndromes formulated by Faegri and van der Pijl^58^. Even though these specific results were consistent with previous studies which supported the pollination syndrome concept^4,12,17,59^, our results suggested that floral traits and pollinators were not significantly associated, and plants were visited by more pollinator species than pollination syndromes predicted (examples see Fig. 4). Therefore, the plant and pollinator species were highly generalized in Yongxing Island, suggesting that pollination syndromes should be used with caution on oceanic islands.

Remote islands provide microcosms for testing the usefulness of pollination syndrome to predict the main pollinator. Plants on islands are faced with an environment where the species numbers of classical pollinator groups, such as bees, butterflies or birds are extremely low^60,61^. One strategy to adapt to a limited number of potential pollinators is the evolution of features promoting generalist pollination, since generalist pollinators are more common and reliable on oceanic islands^46^. Hence, generalized pollination systems may be advantageous to assist plants to expand their ranges into new habitats^62^ and considered a reproductive assurance mechanism evolved primarily on oceanic island environments^31^. In the other side, facing with limited food resources, pollinators may change their behavior to visit flowers with floral traits that they used to avoid when resources are abundant. In Yongxing Island community, most plant species host a taxonomically diverse array of pollinators, and most pollinators visited plant species with different floral syndromes. Therefore, the pollination systems espoused by Faegri and van der Pijl^58^ may be unreliable predictors of floral pollinators on oceanic islands. Similar results were also found in community-level studies in Mediterranean climates of the northern hemisphere^63,64^ and in Tasmania^12^. On tropical islands, pollination by generalist birds is common^46^, and flowering plants are frequently visited by non-flower-specialized birds which do not fit a typical ornithophilous syndrome^24^. Floral traits in most plant species on oceanic island may have the potential for adapting to new conditions under changing pollination environments^65,66^. Pollination syndromes in species from tropical regions are significantly more adjustable accustomed to a changing habitat than in species from other regions^11^. Thus, the pollination syndrome concept, as it is currently defined and used, appears to be a reliable predictor of pollinators primarily for highly specialized systems which are obvious^67^, but it may be not suitable for generalized systems.

To our knowledge, no other studies have examined the relationship between floral traits and pollinators, i.e. testing the pollination syndrome concept in the oceanic island community, even if such data are very valuable^10,24^. In our study, following previous studies^48,68^, we presumed the visitors carried the host plant pollen grains as the potential pollinators (i.e. putative or prospective pollinators), although visitor’s body pollen loads are not necessarily good estimates of pollinator effectiveness in a few plant species^69,70^. Pollen deposition success is the most frequently used proxy for assessing the pollinator’s contribution to plant reproductive success^71,72^. As we did not test the pollination effectiveness of a flower visitor by analyzing visit frequency and pollen deposition on stigmas per visit^69^, pollinators recorded in this study must be regarded as prospective or potential pollinators. However, the ability of carry pollen grains is one necessary condition for animals to become the effective pollinator. We recognize that our conclusions based on field observation and examining insect body pollen loads are imperfect as we only confirmed the potential pollinators to each plant species by excluding the visitors that did not capture pollen grains, and we either did not quantify the efficiency of pollination or investigate the seed set of each plant species for each pollination linkage. This approach is likely to overestimate the degree of generalization. The pollinator species differ in characteristics and behaviors on different plant species that would influence their pollination efficiency, leading to the difference of pollinator’s ability depositing pollen grains on the stigma of plant. Ignoring the difference of pollination efficiency of each pollinator may exaggerate the connectance of plant-pollinator interactions^73^. However, different pollination efficiency may exert different selective pressure on floral traits, and eventually influence the floral evolution together. For instance, floral corolla curvature, floral scents and flora color are all shaped by different pollinator-mediated selection together^74–76^. Also, there are counterbalancing biases towards underestimating generalization and opportunism in pollination interactions. Field surveys are invariably restricted in temporal and spatial dimensions. As pollination interactions vary along these dimensions, surveys may underestimate their complexity. Specialization of the solitary bees is overestimate by the practice of studying pollen collection, and the paucity of records of plants visited for nectar^73^. There may be a widespread unconscious bias to ignore visitors that seem “improper” under the paradigm of pollination syndromes^77^. As approximation, the presence of biases in both directions suggests that observation results may reflect large-scale patterns with reasonable accuracy. As we only investigated one island in this study, we stress that we do not take our results as evidence against the pollination syndrome concept. In fact, we adhere strongly to the vies that many floral traits reflect adaptive response to selection by pollinators^19^. However, we propose that caution is suggested when using pollination syndromes for predicting floral visitors and inferring agents of floral adaption, especially for oceanic island communities. Further research is required to test the applicability in different habitat for understanding the evolution of floral phenotypes.

Even though studies have been focused on understanding the relationships between plant-animal interaction and the evolution of floral traits^1,78,79^, little is known about the relationship between ecological and evolutionary specialization^4^. Our results showed that restrictive flowers were related to higher ecological specialization levels than unrestrictive flowers. This relationship was consistent with the common assumption that restrictiveness flower shape always receives fewer pollinator species^1^ and showed higher ecological specialization. The complex flowers (e.g. restrictive flowers) might reflect the selection by narrower pollinator groups^1,80^, and they were expected to be consistently the most ecologically specialized plants within communities^4^. However, different from Lázaro et al.’s^4^ study, in our community, other floral traits (floral symmetry, flower color, flower clustering and flower size) had no significant effect on the plant ecological specialization. This might be because some animal species which successfully colonize isolated islands tend to broaden their trophic niches, thus interacting with more species than their continental counterparts in order to survive in such low diversity ecosystems^35^, leading to generalization pollination systems in the community. The ecological generalization of plant species is more dependent on the community-context than on specific floral traits^4^. On oceanic islands, plant niche overlap is greater than on mainland, which might be attributed to the relatively smaller number of pollinators in the communities^37^. Plants on oceanic islands usually have high levels of generalization, making them less susceptible to the loss of any particular pollinator species^81^. Meanwhile, pollinators are more dependent on plants on oceanic islands than on mainlands^82^. These pollination system structural features may influence the plants ecological specialization, and may associate with the unavailability of pollination syndromes within the oceanic island communities.

## Conclusions

In summary, our results suggested that using pollination syndrome concept to predict the pollinators of plants may have its limits in the tropical oceanic islands. Plants are visited not only by pollinators that pollination syndromes predicted but also by other pollinator functional groups, a result mostly attributable to the relatively smaller number of pollinator species and highly generalization levels of pollination systems on islands. Thus, the pollination syndrome concept may be unreliable predictors of floral pollinators in oceanic island communities. Restrictive flowers showed more specialization than unrestrictive flowers. But the ecological specialization of plant species was not related to other floral traits in the community, showing that the ecological specialization may be more dependent on the community-context than on specific floral traits^4^. This may be a result of limited pollination in small, isolated and depauperated island systems such as Yongxing Island. Our results supported the viewpoint of Hervías‐Parejo et al.^24^ that pollination syndrome thinking was based on a instable foundation, particularly on islands where decreasing resources can weaken specialization and blur the syndrome boundaries. Receiving more visits from pollinator species of flowers may help plants to adjust their reproductive strategy in response to the pollinator-poor environments, providing opportunity for nonspecialized visitors, which might eventually lead to floral evolution. Our results confirm the validity of the pollination syndrome concept, but also highlight that only using pollination syndrome concept to predict the pollinators of plants is unsuitable on oceanic islands.

## Supplementary information

Supplementary Table 1.
