# Supplementary material for: Plants are visited by more pollinator species than pollination syndromes predicted in an oceanic island community
Source: Sci Rep. 2020 Aug 18;10:13918. doi: 10.1038/s41598-020-70954-7 (PMC7434763; doi:10.1038/s41598-020-70954-7)
Supplement: Supplementary file 1 — Supplementary Table 1. [file 41598_2020_70954_MOESM1_ESM.pdf]

Plants are visited by more pollinator species than pollination syndromes predicted in an oceanic island community

Xiangping Wang[1], Meihong Wen[1], Xin Qian[1], Nancai Pei[2], Dianxiang Zhang[1, *]

[1]Key Laboratory of Plant Resources Conservation and Sustainable Utilization, South China Botanical Garden, Chinese Academy of Sciences, Guangzhou, Guangdong, China

[2]Research Institute of Tropical Forestry, Chinese Academy of Forestry, Guangzhou, China

*Correspondence should be addressed to:

Prof. Dr. Dianxiang Zhang: dx-zhang@scbg.ac.cn

Table S1. Flowering plants and the numbers of their pollinator species to each functional group.

Study species	Apidae	Non-apidae Hymenoptera	Syrphidae	Non-syrphidae Diptera	Butterfly	Hawkmoth	Hemiptera	Passeriformes
Abutilon indicum	1	5						
Bidens pilosa	6	12	2	3	2		1	
Boerhavia diffusa	1	3	1	1				
Bougainvillea spectabilis			1	1	1			
Canavalia maritima	2							
Carica papaya	1					1		
Catharanthus roseus	2	1			1			
Chromolaena odorata	3	6	1	1	2			
Clerodendrum inerme					1			
Cleome viscosa	4	10	3	2	1			
Coccinia grandis	3							
Colubrina asiatica		2						
Cordia subcordata	1							1
Crotalaria pallida					1			
Datura metel	4	3		1		1	1	
Eclipta prostrata	1	2						
Euphorbia atoto	2	4	1	3	2			
Euphorbia cyathophora	5	5	1	5	1			

Euphorbia hirta	2	9	1	3		
Gossypium hirsutum	1					
Guettarda speciosa						4
Herissantia crispa	1					
Ipomoea obscura	3				1	
Ipomoea pescaprae	5	4	1	1		
Ixora chinensis					1	
Lantana camara	1	2			3	1
Macroptilium atropurpureum	1				1	
Messerschmidia argentea	5	3	2	6	1	1
Mimosa pudica	1					
Morinda citrifolia	2		1	1		2
Passiflora foetida	4	2		1		
Physalis minima	3	4		1		
Phyla nodiflora		3	1	1	1	
Pisonia grandis	4	8	1	2	2	
Portulaca grandiflora	1			1		
Portulaca oleracea	3	6		4	2	
Rhynchosia minima	1	1			1	
Ricinus communis	1					1
Scaevola taccada	6	14		3		3

Senna occidentalis	3				1		
Sesbania cannabina	1	3			2		
Sesuvium portulacastrum	3	2		1			
Sida alnifolia	3						
Solanum photeinocarpum	4	1	1				
Stachytarpheta jamaicensis	2	2	1	1	1		
Suriana maritima	2						
Terminalia catappa	2	3		3	2		
Tribulus cistoides	4	9	3	4	4		
Trianthema portulacastrum	4	12	2	3	2	2	
Tridax procumbens	6	17	3	7	8	2	1
Triumfetta procumbens	1	1					
Vernonia cinerea		2	1		2		
Vigna marina	1	2			4		
Wedelia biflora	6	17	2	10	9		1
Wedelia trilobata	6	12	2	5	9		
